# Editorial: Endo- and transcytotic pathways at the brain barriers

**DOI:** 10.3389/fddev.2024.1415104

**Published:** 2024-05-06

**Authors:** Michelle A. Erickson, Morten S. Nielsen

**Affiliations:** ^1^ Geriatric Research Education and Clinical Center (GRECC), VA Puget Sound Healthcare System, Seattle, WA, United States; ^2^ Department of Medicine- Division of Gerontology and Geriatric Medicine, University of Washington School of Medicine, Seattle, WA, United States; ^3^ Department of Biomedicine, Aarhus University, Aarhus, Denmark

**Keywords:** blood-brain barrier, endocytosis, transcytosis, antibodies, bispecific antibodies, biologics, insulin, transferrin receptor

Brain barriers protect and support the central nervous system, but also impede drug delivery to the brain. Vesicular transport is a predominant mechanism by which substances can cross brain barriers, via processes such as receptor-mediated transcytosis (RMT). The articles published in this thematic issue highlight important aspects of RMT that have been leveraged to facilitate the delivery of substances to the brain. They also point to many of the challenges that have been realized, and in some cases, overcome for CNS delivery of biologics.

The mini-review article by Baghirov presents an insightful and critical analysis of vascular blood-brain barrier (BBB) transport mechanisms of biologics. The article begins by noting that even substances that are not necessarily targeted to the CNS, such as larger Adeno-associated viruses or extracellular vesicle biologics, may bind proteins in the circulation which then facilitate their interactions with brain barriers, resulting in improved uptake of the entire particle or particle cargo into the brain. This article also describes some of the nuances pertaining to usage of the transferrin receptor and low-density lipoprotein receptor-related protein-1 (LRP-1), as well as some solute carriers and their use as CNS delivery shuttles.

The full-length review article by Pardridge provides an in-depth analysis of CNS antibody therapeutics. In this review, a detailed overview is provided on antibodies that have been evaluated and/or approved for treatment of CNS diseases including Multiple Sclerosis, Brain Cancer, and Alzheimer’s disease. Key considerations on methods of measuring and interpreting brain entry of antibodies are discussed, as are BBB transporters that have been studied for their utility in CNS drug delivery. Bispecific antibodies are those which have two distinct binding domains, and in the case of CNS drug delivery, are typically engineered to bind a BBB transporter shuttle at one site, and the CNS protein target on the second site. The engineering, cell biology, pharmacology, and therapeutic testing of bispecific antibodies is also discussed in detail.

The Brief Research Report by Fukatsu et al. used *in vitro* cell models to test the mechanisms by which pabinafusp alfa crosses the BBB and enters brain cells. Pabinafusp alfa is an anti-mucopolysaccharidosis II (MPSII) drug comprised of iduronate-2-sulfatase genetically fused with an anti-transferrin receptor (TfR) antibody to improve CNS delivery. MPS II patients have a mutation in the IDS gene that encodes iduronate-2-sulfatase (I2S), resulting in a buildup of glycosaminoglycans throughout the body, including in the brain. Therapies for MPSII are those that replace the deficient I2S enzyme, but delivering therapeutic enzymes to the brain has been a historic challenge to treating MPSII and related enzyme deficiencies. Pabinafusp alfa was recently approved in Japan for the treatment of MPSII (Giugliani et al., 2021). Mannose-6-phosphate is a common carbohydrate modification found on lysosomal enzymes such as I2S (Sleat et al., 2006), and lysosomal enzymes, viruses/viral glycoproteins, and other substances can cross the intact BBB and be taken up by cells via the mannose-6-phosphate receptor (Mbemba et al., 1994; Urayama et al., 2008; Dohgu et al., 2012). In their study, the authors tested using pharmacologic inhibition studies whether the transferrin receptor or mannose-6-phosphate receptor predominantly mediated BBB transport and brain cell uptake. They showed, using cell lines of human brain endothelial cells, pericytes, astrocytes, and neurons that the transferrin receptor mediated BBB transport and cellular uptake of pabinafusp alfa, whereas the unconjugated I2S enzyme was transported and taken up by the mannose-6-phosphate receptor. The demonstrated predominance of the transferrin receptor in mediating uptake offers insight into the factors regulating biodistribution of pabinafusp alfa in the brain.

The original research article by Pemberton et al. used chemical inhibitors of clathrin and caveolae-mediated vesicular pathways to delineate the predominant routes of insulin binding to its receptor and transporter, which were recently shown to be distinct entities (Rhea et al., 2018). Clathrin inhibition in isolated brain microvessels increased insulin binding, and when given *in vivo* increased the overall binding of S961, an insulin receptor antagonist that binds brain endothelial cells but is not transported across the BBB. Caveolin inhibition did not have effects on insulin binding to brain microvessels. These data suggest that at the BBB, clathrin regulates insulin receptor binding. In contrast, caveolin inhibition preferentially inhibited insulin transport across the BBB, although this effect was not widespread but specifically limited to the hypothalamus. Results from this study thus offer important insight into the endocytic mechanisms regulating cerebrovascular insulin binding, and insulin uptake into the hypothalamus-an important regulatory site for insulin’s metabolic effects (Belsham and Dalvi, 2020).

The regulation of vesicular pathways at brain barriers remains an elusive, yet attractive area of study. Advances in molecular tools that can label components of endocytic and transcytotic machinery, as well as advanced microscopic techniques that facilitate visualization at high-resolution will likely advance our understanding of vesicular trafficking across brain barriers. The publications in this thematic issue highlight many of the approaches that have been taken to optimize the design of biologics that leverage receptor-mediated transcytosis to enter the brain, and to understand the cellular and molecular mechanisms that regulate BBB transport of substances and subsequent engagement with their targets. Further endeavors that aim to expand knowledge on existing transporter systems, and to discover and characterize new transporters could provide improved strategies for drug delivery to the CNS.
